# The Current Landscape of Clinical Studies Focusing on Thyroid Cancer: A Comprehensive Analysis of Study Characteristics and Their Publication Status

**DOI:** 10.3389/fendo.2020.575799

**Published:** 2020-11-20

**Authors:** Yihao Liu, Bin Li, Qiuyi Zheng, Jia Xu, Jie Li, Fenghua Lai, Bo Lin, Sui Peng, Weiming Lv, Haipeng Xiao

**Affiliations:** ^1^ Clinical Trials Unit, The First Affiliated Hospital of Sun Yat-sen University, Guangzhou, China; ^2^ Department of Endocrinology, The First Affiliated Hospital of Sun Yat-sen University, Guangzhou, China; ^3^ Department of Breast and Thyroid Surgery, The First Affiliated Hospital of Sun Yat-sen University, Guangzhou, China

**Keywords:** clinical studies, thyroid cancer, targeted therapy, publication status, ClinicalTrials.gov

## Abstract

**Background:**

A better understanding of the current characteristics of clinical trials on thyroid cancer (TC) is important to improve trial designs and identify neglected areas of research. However, there is a lack of a thorough understanding of the clinical studies on TC. Therefore, this study aimed to present a comprehensive overview of clinical trials on TC based on the ClinicalTrials.gov database and evaluate their publication status.

**Methods:**

We searched for TC-related clinical studies registered in the ClinicalTrials.gov database before December 2018 by using the keyword “thyroid cancer” and assessed the characteristics of the included trials. We searched the publication status of primary completed studies in PubMed and Google Scholar.

**Results:**

A total of 450 studies were identified for analysis, including 333 (74.0%) interventional studies and 117 (26.0%) observational studies. Interventional studies about TC were commonly non-randomized (67.6%), single-arm (55.6%), single-center (76.3%), and early-phase (60.0%) trials. The major category for which studies were performed was for target drug-related therapy (53.6%). In addition, 57.0% of the primary completed interventional studies were published. The published studies were more commonly primary completed studies after 2010 and used randomization and were less commonly designed as single-arm studies and were conducted in the USA/Canada, compared to non-published studies (*P* < 0.05 for all). The median time from primary completion to publication was 46.5 months, and the time decreased to 36.5 months after 2010. Studies conducted in the USA/Canada [odds ratio (OR) = 9.43, *P* = 0.020] and multi-center studies (OR = 6.55, *P* = 0.021) significantly increased the potential of publication in high-impact journals.

**Conclusions:**

High-quality, randomized phase 3 trials regarding TC are still insufficient. Therefore, more efforts are needed to improve the treatment of poor prognostic TC and timely publication.

## Introduction

The incidence of thyroid cancer (TC) had increased rapidly worldwide in the last few decades (1). Approximately 50% of newly diagnosed TC cases are papillary thyroid microcarcinoma (PTMC) (2), defined as papillary TC ≤1 cm in diameter, the vast majority of which are indolent in nature and do not result in death. The 2015 American Thyroid Association (ATA) guidelines recommended active surveillance as an alternative treatment for low-risk PTMC (3). Nonetheless, the potential risk and benefit of active surveillance are still unclear.

The prognosis of TC is generally excellent, owing to its natural biological behavior (4). However, the treatment of TC with a relatively poor prognosis, such as advanced or radioiodine-refractory differentiated thyroid cancer (DTC), medullary thyroid cancer (MTC), and anaplastic thyroid cancer (ATC), is a serious challenge (3). Despite advances in the understanding of cancer biology and the development of molecular-targeted drugs, there has been only a slight improvement in the outcomes for those patients. A better understanding of the current composition and characteristics of clinical trials on TC is important to improve the trial design and identify neglected areas of research.

Clinical trials, especially well-designed randomized clinical trials, have been the foundation of evidence-based medicine and the driving force in the development of medicine. In 2004, the International Committee of Medical Journal Editors (ICMJE) advocated that clinical trials should be registered in a public registry before recruiting patients to ensure transparency of the process (5, 6). The ClinicalTrials.gov database is a publicly available, worldwide, trial registry, developed and maintained by the National Library of Medicine (NLM) for the National Institutes of Health (NIH). Currently, the ClinicalTrials.gov database provides the most comprehensive information about ongoing and completed clinical studies worldwide (7, 8).

Although previous studies have evaluated a subset of oncologic-based clinical trials, none have focused exclusively on TC (8, 9). Currently, physicians still lack a thorough understanding of clinical studies on TC. Thus, given the need for better and more efficient clinical trials, it is necessary to identify new developments and to maintain the current information to guide future trial design. Moreover, registration is expected to improve transparency in the process of conducting and reporting trials. However, the characteristics associated with publication and timeliness of publication have not been studied for TC. Therefore, we aimed to present a comprehensive landscape of TC-related studies based on the ClinicalTrials.gov database and evaluate the publication status of these studies.

## Methods

### Searching the ClinicalTrials.gov Database

We searched the ClinicalTrials.gov database on December 31, 2018, using the search term “thyroid cancer.” The search date was limited from 1st January 2004 to 31st December 2018. All available results were downloaded in the form of xml files. Afterward, all the data were imported into a database to facilitate further data cleaning, classification, and management. Studies under withdrawn, unknown, terminated, and expanded access statuses were excluded. After reviewing the trial summary, studies that did not include TC were also excluded. The remaining studies were selected for further manual classification analysis. This study was approved by the Research Ethics Committee of the First Affiliated Hospital of Sun Yat-sen University.

### Variables of the Registered Studies

According to the information given on the ClinicalTrials.gov database, the following variables of the registered studies were categorized by two investigators (YL and QZ) independently: disease [only TC or multi-cancer (multi-cancer means that the study also recruit patients with other kind of cancer, but not limited to thyroid cancer)], age (only adults/adults and children), TC subtypes (DTC/MTC/ATC/advanced or radioiodine-refractory DTC/unclassified advanced or metastasis TC/unclassified TC/others), treatment used in interventional studies (targeted drugs/targeted drugs and other therapy/radiotherapy, including I_131_/immunotherapy/chemotherapy/others), purpose of the interventional studies (treatment/basic science/health service or preventive/supportive care/screening/diagnostic), study design of observational studies (case-only/cross-sectional/case-control/cohort), study design of interventional trials (single group/parallel/factorial/crossover/sequential), countries where the study was performed (the USA or Canada/European/Asian/others), centers (single-center or multi-center), and funder (industry/NIH/others). If an industry was listed as the lead funder, the trial was classified as being funded by the industry. When NIH was listed as the lead funder, the trial was considered as NIH-funded (10). The time to primary completion was defined as the time from the start of the study to completion of the primary endpoint. The study duration was defined as the time from the start of the study to completion of the study.

### Searching for the Publication Status

Two investigators (YL and BL) independently searched for peer-reviewed publications of studies under primary completion by using a standardized strategy. The “publications” field in the ClinicalTrials.gov database was reviewed to search for potentially matching publications. We then searched PubMed and Google Scholar by using NCT numbers in all the fields. Publication was confirmed by matching the study characteristics in the ClinicalTrials.gov database with the description in the manuscript. The earliest article reporting the primary outcome results was chosen if multiple publications were obtained from a single study. Study protocols, commentaries, interim analysis, and other non-relevant publication types were excluded. A third investigator (JX) independently reconfirmed and conducted a publication search for the studies that were found to be unpublished by the first two investigators. Differences were resolved by consensus. For each published article, the published date, study design, sample size, country, primary outcome (negative or positive), and impact factor (IF) were collected. The search for publication status was updated and finalized by April 1, 2019.

### Statistical Analysis

The number (percentage) for categorical variables and median [interquartile range (IQR)] for continuous variables were calculated. Fisher’s exact tests were used to compare the categorical variables, while the Mann-Whitney U tests were used to compare the continuous variables. Cox regression analysis was performed to analyze the factors influencing the time to publication. The hazard ratios (HRs) and 95% confidence intervals (CIs) were calculated for the factors. The multivariate model included each variable associated with *P* < 0.05 on univariate analysis. The time to publication was estimated by using the Kaplan-Meier method. As the completed studies need enough time to be published, primary studies completed after January 1, 2017, were excluded from the Cox regression analysis. Logistic regression analysis was performed to analyze the factors influencing publication in high-impact factor (≥10) journals. The odds ratios (ORs) and 95% CIs were calculated for the factors.

All statistical tests were performed using Stata/MP version 14.0 (Stata Corporation LP, College Station, TX, USA), and a two-sided *P* < 0.05 was considered statistically significant.

## Results

### Characteristics of the Registered Studies

**Figure 1:**
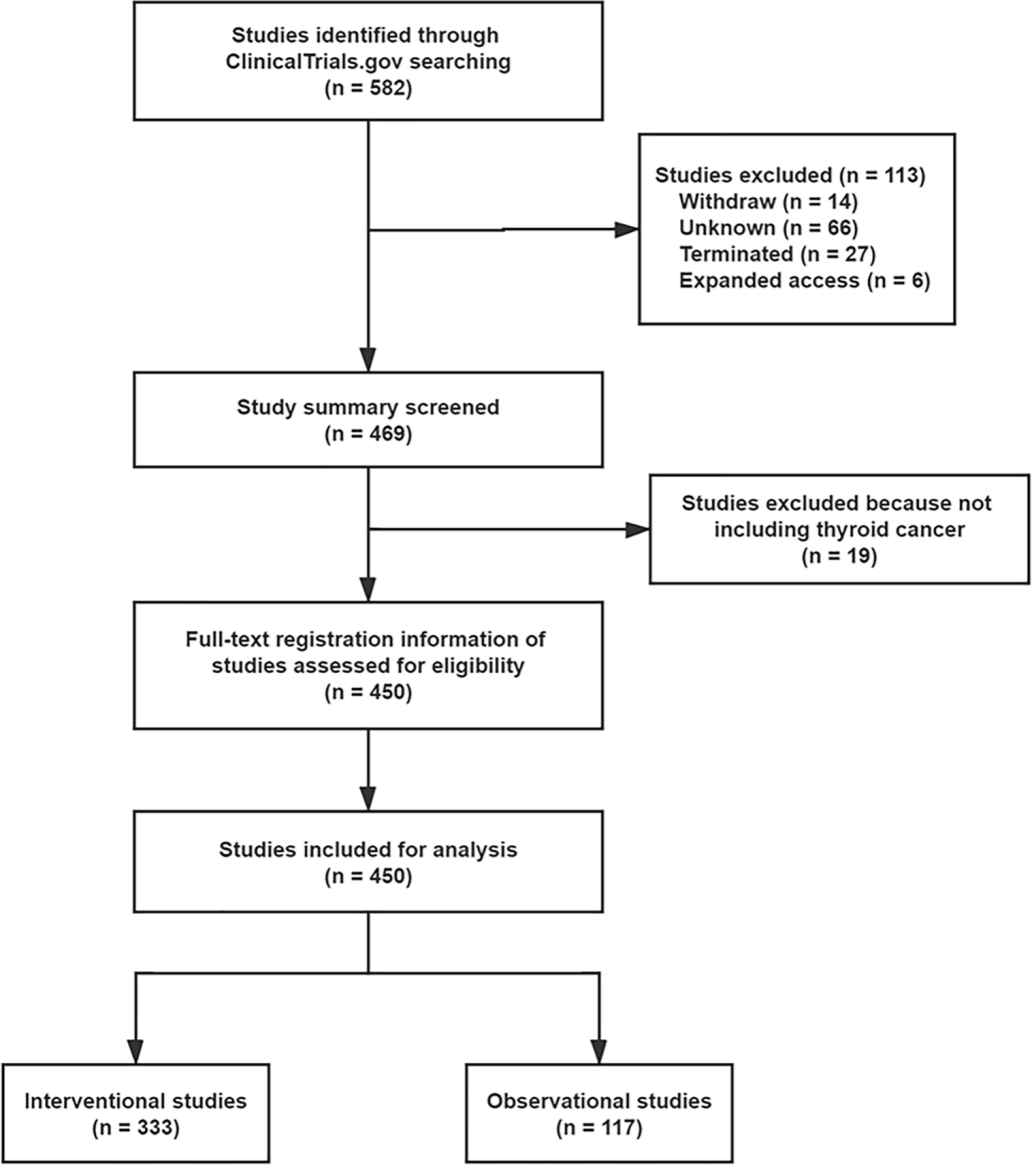
Study selection flow chart.

**Figure 2:**
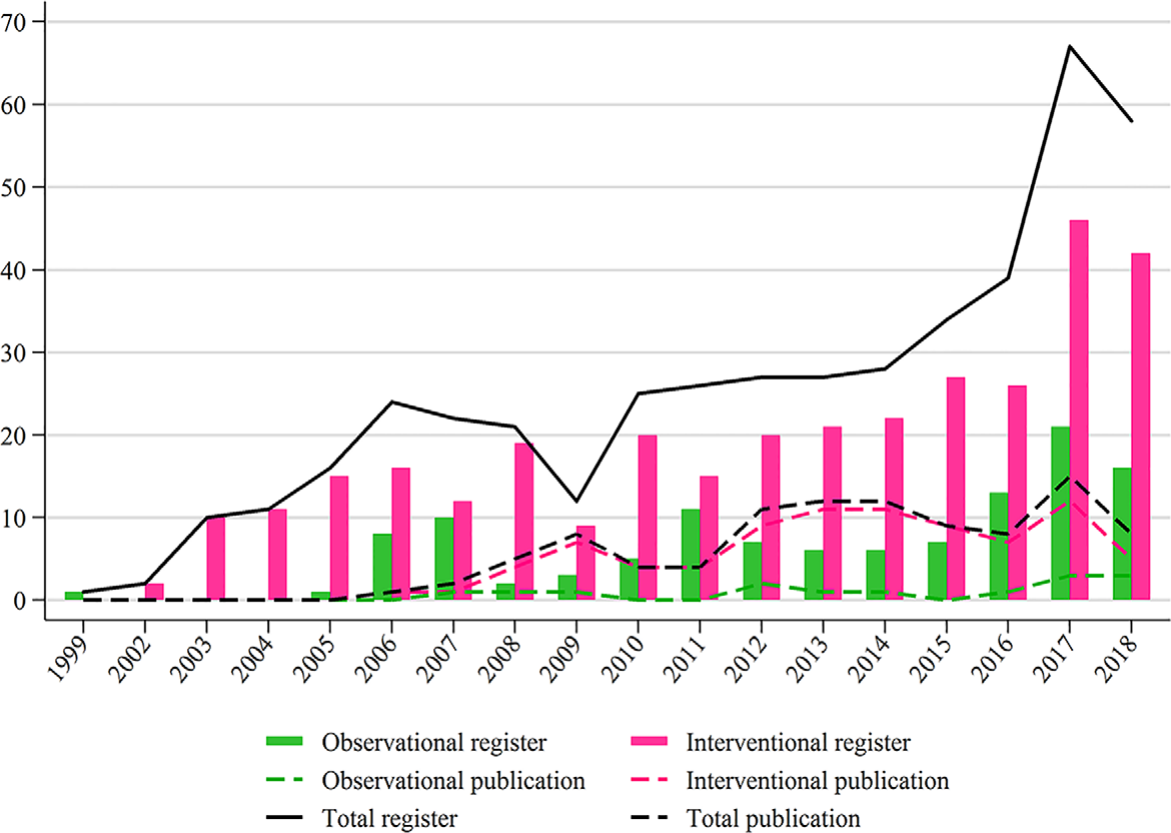
Study distributions of register and publication for observational and interventional studies according to the registered or published year.

A total of 582 registered studies were identified, and 132 studies that were under the withdrawn, unknown, terminated, and expanded access statuses, and did not include TC, were excluded (**Figure 1**) . Finally, 450 studies were evaluated for analysis, including 333 (74.0%) interventional studies and 117 (26.0%) observational studies. The distribution of interventional and observational studies according to the registered time is summarized in **Figure 2**. Overall, the number of registered studies increased over the years, and the number of interventional studies increased more rapidly. More than 20 interventional studies were registered every year after 2012, and the number increased by more than 40 in the last 2 years.

The characteristics of the included interventional and observational studies are summarized in **Table 1**. Compared to observational studies, interventional studies included fewer children; included mostly only TC; had fewer registries after patient recruitment, a smaller sample size, more results available, and more publications; and were more often conducted in the USA/Canada, more often multi-center, and more often funded by the NIH/industry (*P* < 0.05). A total of 35.3% of the interventional trials focused on TC with poor prognosis, while 71% of observational studies were performed for unclassified TC. Among the interventional studies, studies for treatment and diagnosis accounted for 79 and 12%, respectively. In addition, 31 studies (9%) were registered for supportive care, health or preventive service, screening, and basic science. Among the studies for treatment, the major study category was for target drug-related therapy (53.6%). Studies for radiotherapy, chemotherapy and immune therapy accounted for 4.6, 3.8, and 4.6%, respectively.

**Table 1:** Characteristics of interventional and observational studies.

	Interventional (n = 333)	Observational (n = 117)	*P*-values
Age			<0.001
Adults and children	37 (11.1)	35 (29.9)	
Only adults	296 (88.9)	82 (70.1)	
Disease			0.015
Multi-cancer	86 (25.8)	17 (14.5)	
Only TC	247 (74.2)	100 (85.5)	
TC subtypes of only TC studies			<0.001
Unclassified TC	110 (44.5)	71 (71.0)	
Poorer prognostic TC	87 (35.3)	14 (14.0)	
MTC	21 (8.5)	9 (9.0)	
ATC	12 (4.9)	3 (3.0)	
Unclassified advanced or metastasis TC	34 (13.8)	0 (0.0)	
Advanced or radioiodine-refractory DTC	20 (8.1)	2 (2.0)	
Unclassified DTC	44 (17.8)	15 (15.0)	
Others ^a^	6 (2.4)	0 (0.0)	
Sample size			<0.001
<=50	164 (49.2)	23 (19.7)	
51-100	63 (18.9)	16 (13.7)	
101-200	51 (15.3)	21 (17.9)	
>200	53 (15.9)	56 (49.9)	
NA	2 (0.6)	1 (0.9)	
Registered after study start			<0.001
No	133 (39.9)	20 (17.1)	
Yes	200 (60.1)	97 (82.9)	
Study design			
Single group	186 (55.9)	–	
Parallel	132 (39.6)	–	
Sequential	4 (1.2)	–	
Factorial	3 (0.9)	–	
Crossover	7 (2.1)	–	
Case-only	–	18 (15.4)	
Cross-sectional	–	4 (3.4)	
Case-control	–	16 (13.7)	
Cohort	–	64 (54.7)	
NA	1 (0.3)	15 (12.8)	
Time series			
Retrospective	0 (0.0)	21 (17.9)	
Cross-sectional	0 (0.0)	9 (7.7)	
Prospective	333 (100.0)	80 (68.4)	
NA	0 (0.0)	7 (6.0)	
Primary completed			0.669
No	160 (48.0)	59 (50.4)	
Yes	173 (52.0)	58 (49.6)	
Duration of primary completion (mo.)	41.3 (23.9, 60.0)	28.4 (12.5, 72.0)	0.191
Results of primary completed studies			<0.001
No available results	126 (72.8)	57 (98.3)	
Has results	47 (27.2)	1 (1.7)	
Study completion			0.829
Ongoing	182 (54.7)	62 (53.0)	
Completed	151 (45.3)	55 (47.0)	
Study duration (mo.)	49.6 (29.0, 74.2)	35.4 (21.0, 89.7)	0.103
Publication			0.002
No publication	245 (73.6)	102 (87.2)	
Publication	88 (26.4)	15 (12.8)	
Time from primary completion to publication	24.0 (11.2, 38.2)	20.9 (12.0, 36.6)	0.911
Country			0.014
US/Canada	172 (51.7)	49 (41.9)	
European	55 (16.5)	26 (22.2)	
Asian	62 (18.6)	31 (26.5)	
Others	37 (11.1)	5 (4.3)	
NA	7 (2.1)	6 (5.1)	
Center			0.021
Single-center	254 (76.3)	106 (90.6)	
Multi-center	72 (21.6)	5 (4.3)	
NA	7 (2.1)	6 (5.1)	
Funder			<0.001
Industry	112 (33.6)	10 (8.5)	
NIH	62 (18.6)	18 (15.4)	
Others	159 (47.7)	89 (76.1)	
Purpose			
Treatment	261 (78.4)	–	
Diagnostic	41 (12.3)	–	
Supportive care	16 (4.8)	–	
Health service or preventive	12 (3.6)	–	
Screening	1 (0.3)	–	
Basic Science	2 (0.6)	–	
Treatment			
Target drugs	135 (51.7)	–	
Targeted drugs and other therapy	21 (8.0)		
Radiotherapy	12 (4.6)	–	
Chemotherapy	10 (3.8)	–	
Immune therapy	12 (4.6)	–	
Others ^b^	71 (27.2)	–	
Phase			
Phase 1	59 (17.7)	–	
Phase 1/2 or 2	141 (42.3)	–	
Phase 2/3 or 3	33 (9.9)	–	
Phase 4	9 (2.7)	–	
NA	91 (27.3)	–	
Randomization			
No	225 (67.6)	–	
Yes	108 (32.4)	–	
Blind			
Open label	260 (78.1)	–	
Blind	21 (6.3)	–	
NA	52 (15.6)	–	

DTC, differentiated thyroid cancer; MTC, medullary thyroid cancer; ATC, anaplastic thyroid cancer; NA, not available.

Others ^a^ includes poorly differentiated TC, BRAF mutant TC, non-medullary TC, et al.

Others ^b^ includes endocrine therapy, operation, and other drugs (such as antibiotics, antidiabetic drugs, narcotic, et al.).

Regarding the study design, interventional studies were mostly non-randomized (225, 67.5%), open-label (260, 78.1%), single-group (186, 55.9%), single-center (254, 76.3%), and early-phase (200, 60.0%). Only 33 phase 2/3 or 3 trials were registered, accounting for 9.9% of all interventional studies. A total of 54.7% of observational studies were cohort studies.

Among the interventional studies, 173 (52.0%) were primary completed studies, and 22 (12.8%) were still ongoing considering the long-term outcomes. The median duration for primary completion was 40.0 months (IQR: 22.0–57.2).

### Publication Status of Primary Completed Studies

Only 15 registered observational studies were published. Among 149 primary completed interventional studies before January 1, 2017, 84 (57.0%) were published by April 1, 2019. Since 2012, approximately ten interventional studies were published every year (**Figure 2**).

The characteristics of published and unpublished interventional studies are shown in **Table 2**. Among the published studies, more were primary completed studies after 2010 and were randomized, while fewer were single-arm and conducted in the USA/Canada, compared to unpublished studies (*P* < 0.05 for all).

**Table 2:** Characteristics of interventional studies which had primary completed.

	Total (n = 149)	No publication (n = 65)	Published (n = 84)	*P* values
Duration of primary completion	40.0 (22.5, 56.0)	46.5 (25.7, 60.0)	34.2 (20.5, 54.0)	0.062
Study duration	51.0 (30.0, 85.0)	51.0 (33.0, 86.0)	50.8 (25.0, 82.0)	0.477
Study status				0.187
Ongoing	10 (6.7)	2 (3.1)	8 (9.5)	
Completed	139 (93.3)	63 (96.9)	76 (90.5)	
Year of primary completion				0.043
2000–10	59 (39.6)	32 (49.2)	27 (32.1)	
2011–16	90 (60.4)	33 (50.8)	57 (67.9)	
Registered after study start				0.356
No	41 (27.5)	15 (23.1)	26 (31.0)	
Yes	108 (72.5)	50 (76.9)	58 (69.0)	
Sample size				0.722
<=50	77 (51.7)	35 (53.8)	42 (50.0)	
51–100	35 (23.5)	15 (23.1)	20 (23.8)	
101–200	35 (23.5)	13 (20.0)	22 (26.2)	
NA	2 (1.3)	2 (3.1)	0 (0.0)	
Age				0.299
With child	16 (10.7)	9 (13.8)	7 (8.3)	
Only adult	133 (86.7)	56 (86.2)	77 (91.7)	
Condition				0.090
Multi-cancer	39 (26.2)	22 (33.8)	7 (20.2)	
Only TC	110 (73.8)	43 (66.2)	67 (79.8)	
TC type of only TC studies				0.045
Unclassified TC	51 (46.4)	24 (55.8)	27 (40.3)	
Poorer prognostic TC	41 (37.2)	12 (27.9)	29 (43.2)	
MTC	10 (9.1)	0 (0.0)	10 (14.9)	
ATC	2 (1.8)	1 (2.3)	1 (1.5)	
Unclassified advanced or metastasis TC	15 (13.6)	5 (11.6)	10 (14.9)	
Advanced or radioiodine-refractory DTC	14 (12.7)	6 (14.0)	8 (11.9)	
Unclassified DTC	14 (12.7)	4 (9.3)	10 (14.9)	
Others	4 (3.6)	3 (7.0)	1 (1.5)	
Purpose				0.057
Treatment	121 (81.2)	48 (73.8)	73 (86.9)	
Others	28 (18.8)	17 (26.2)	11 (13.1)	
Intervention of treatment				0.710
Target drugs	59 (48.8)	22 (45.8)	37 (50.7)	
Others	62 (51.2)	26 (54.2)	36 (49.3)	
Phases				0.054
Phase 1	29 (19.6)	18 (27.7)	11 (13.3)	
Phase 1/2 or 2	60 (40.5)	23 (35.4)	37 (44.6)	
Phase 2/3 or 3 or 4	25 (16.9)	7 (10.8)	18 (21.7)	
NA	34 (23.0)	17 (26.2)	17 (20.5)	
Study design				0.018
Single group	94 (63.1)	48 (73.8)	46 (54.8)	
Two or more groups	55 (36.9)	17 (26.2)	38 (45.2)	
Randomization				0.021
No	101 (67.8)	51 (78.5)	50 (59.5)	
Yes	48 (32.2)	14 (21.5)	34 (40.5)	
Blind				0.233
Open label	116 (77.9)	54 (83.1)	62 (73.8)	
Blind	33 (22.1)	11 (16.9)	22 (26.2)	
Country				<0.001
USA/Canada	80 (53.7)	47 (72.3)	33 (39.3)	
European	27 (18.1)	8 (12.3)	19 (22.6)	
Asian	24 (16.1)	5 (7.7)	19 (22.6)	
Others	18 (12.1)	5 (7.7)	13 (15.5)	
Center				0.116
Single-center	99 (66.4)	48 (73.8)	51 (60.7)	
Multi-center	50 (33.6)	17 (26.2)	33 (39.3)	
Funder				0.053
Industry	38 (25.5)	23 (35.4)	15 (17.9)	
NIH	49 (32.9)	19 (29.2)	30 (35.7)	
Others	62 (41.6)	23 (35.4)	39 (46.4)	

Regarding the completed studies need enough time to be published, studies whose primary completed date was after 1 January 2017 were excluded from the publication analysis.

**Figure 3:**
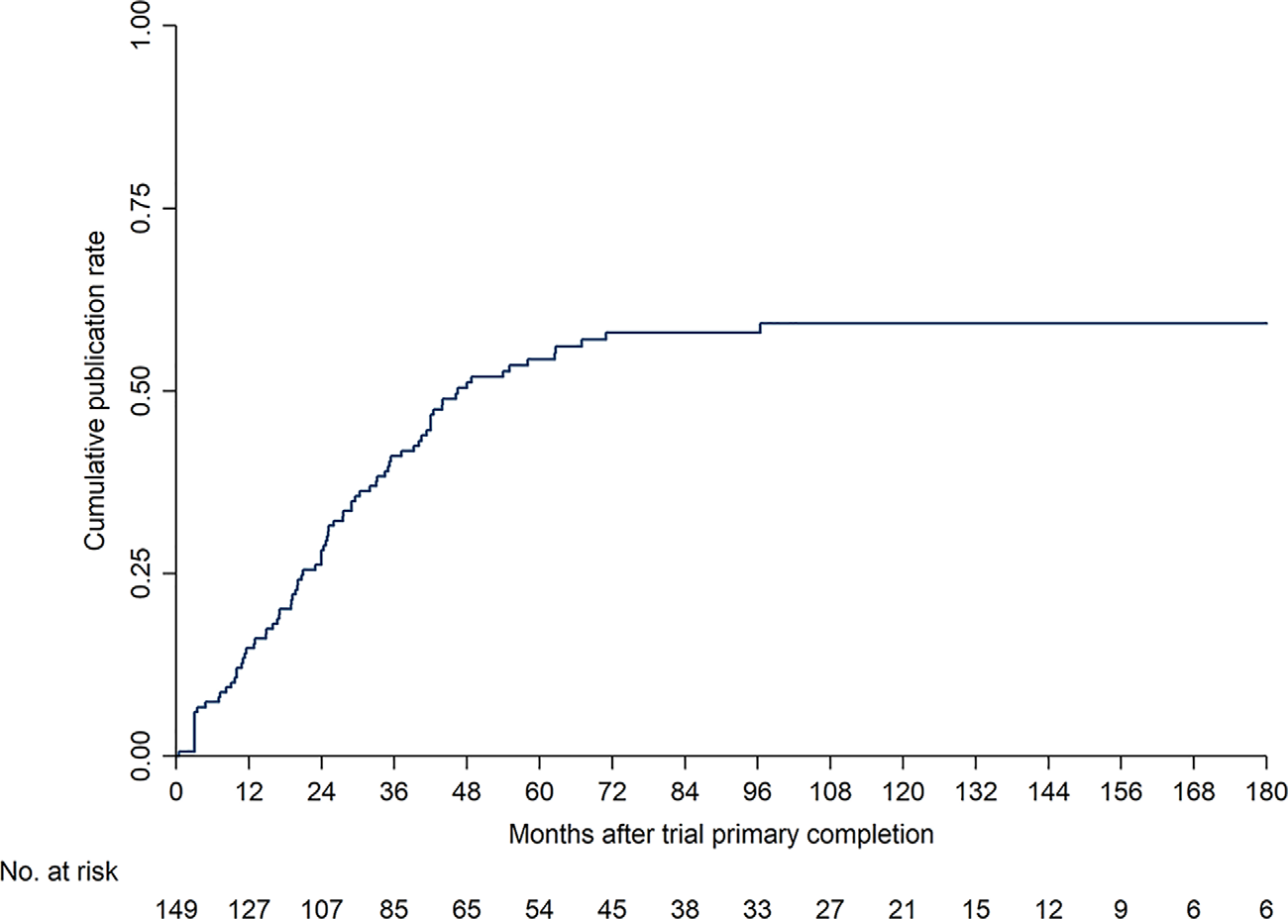
Cumulative publication rate curve from trial primary completion to publication.

The median time to publication was 46.5 months (95% CI: 37.16–96.46 months), and the 1, 2, 3, and 5-year cumulative publication rates were 14.8, 28.2, 41.1, and 54.4%, respectively (**Figure 3**). The factors influencing the time to publication from primary completion of interventional studies is shown in **Table 3**. The year of primary completion, study phases, funder, study design, randomization, and country significantly influenced the time to publication on univariate Cox regression analysis. However, on multivariate Cox analysis, only the year of primary completion and country were significant factors. Primary completed studies after 2010 were more often published on time, and the median time to publication was 35.2 months (HR = 1.85, 95% CI = 1.10–3.12, *P* = 0.021). Asian studies had lesser time to publication, with a median of 29.0 months, compared to studies conducted in the USA/Canada (HR = 2.23, 95% CI = 1.02–4.90, *P* = 0.046).

**Table 3:** Cox regression analysis for time to publication from primary completion of interventional studies.

	Median time to publication (mo.) *	Unadjusted HR (95% CI)	*P*-values	Adjusted HR (95% CI)	*P*-values
Duration of primary completion (mo.)	–	0.99 (0.99, 1.00)	0.152		
Study duration (mo.)	–	1.00 (0.99, 1.00)	0.640		
Year of primary completion					
00–10	NA	1.00		1.00	
11–16	35.2	2.06 (1.29, 3.29)	0.003	1.85 (1.10, 3.12)	0.021
Registered after study start					
No	40.0	1.00			
Yes	62.5	0.68 (0.43, 1.09)	0.110		
Sample size					
<=50	46.5	1.00			
51–100	54.0	1.03 (0.60, 1.75)	0.921		
>100	42.0	1.19 (0.71, 2.00)	0.510		
Age					
With child	NA	1.00			
Without child	44.0	1.55 (0.71, 3.36)	0.271		
Only TC					
No	NA	1.00			
Yes	42.5	1.62 (0.95, 2.76)	0.076		
Purpose					
Others	NA	1.00			
Treatment	42.0	1.74 (0.92, 3.28)	0.087		
Phases					
Phase 1	NA	1.00		1.00	
Phase 2	42.0	2.08 (1.06, 4.09)	0.033	1.70 (0.84, 3.43)	0.139
Phase 3/4	34.5	2.64 (1.24, 5.60)	0.011	0.92 (0.33, 2.58)	0.870
NA	62.5	1.55 (0.73, 3.28)	0.253	0.52 (0.20, 1.37)	0.187
Funder					
NIH	NA	1.00		1.00	
Industry	42.5	1.72 (0.93, 3.21)	0.086	1.04 (0.49, 2.22)	0.910
Others	37.2	1.91 (1.05, 3.47)	0.034	1.29 (0.56, 2.96)	0.545
Study design					
Single group	96.5	1.00		1.00	
Two or more groups	35.0	1.61 (1.04, 2.48)	0.031	1.22 (0.57, 2.62)	0.610
Randomization					
No	71.0	1.00		1.00	
Yes	35.0	1.61 (1.04, 2.49)	0.034	1.40 (0.57, 3.41)	0.460
Blind					
Open label	48.0	1.00			
Blind	33.0	1.4 (0.86, 2.29)	0.173		
Country					
USA/Canada	NA	1.00		1.00	
European	42.0	2.03 (1.15, 3.58)	0.014	1.87 (0.88, 3.97)	0.102
Asian	29.0	2.89 (1.63, 5.12)	<0.001	2.23 (1.02, 4.90)	0.046
Others	34.5	2.04 (1.07, 3.90)	0.030	1.79 (0.75, 4.30)	0.192
Center					
Single-center	55.0	1.00			
Multi-center	40.0	1.36 (0.88, 2.10)	0.172		

NA, not available, the median of time to publication was not reached.

* means median time to publication from primary completion of interventional studies.

Among the published interventional studies, 23 (26.7%) were published in journals with an IF ≥10. As shown in **Supplementary Table S1**, the studies published in high-impact journals were more often regarding treatment, multi-center studies, conducted in the USA/Canada, and funded by industry. After multivariate logistic regression analysis, compared to studies being conducted in the USA/Canada, studies performed in European (OR = 0.09, *P* = 0.020) and Asian (OR = 0.11, *P* = 0.034) countries significantly decreased the probability of being published in journals with an IF ≥10. In contrast, multi-center studies (OR = 6.55, *P* = 0.021) significantly increased the probability of being published in journals with an IF ≥10 (**Supplementary Table S2**).

## Discussion

To our knowledge, the current study is the most comprehensive assessment of clinical trial characteristics of studies on TC and their publication status. Our results showed that clinical trials for TC were commonly non-randomized, single-arm, single-center trials, predominantly performed as phase I or II studies. Both interventional and observational studies kept increasing in recent years. Current interventional studies were focused on targeted drug-related therapy, especially for patients with poor-prognosis TC subtypes. Only 57.0% of the primary completed interventional studies were published, and the median time to publication was 46.5 months. Encouragingly, there was an improvement in the time to publication.

Targeted therapies had been introduced and brought survival benefit to patients with different cancers over the past decades (11–13). In the current study, 59.7% of the interventional studies focused on targeted therapies for TC. Although numerous clinical studies have evaluated distinct targeted drugs, only four drugs have unequivocally shown their effectiveness in terms of improvement in the progression-free survival in randomized, placebo-controlled, phase III clinical trials: sorafenib (NCT00984282) (14) and lenvatinib (NCT01321554) (15) for radioiodine-refractory DTC and vandetanib (NCT00410761) (16) and cabozantinib (NCT00704730) (17) for MTC. Nonetheless, none of the targeted drugs have shown significant benefit in OS, except for a subgroup of patients receiving lenvatinib or cabozantinib (17, 18). Regardless of the limited treatment effect, a high rate of adverse events was observed in patients who received targeted therapy (19, 20). Immunotherapy, including immune cells and checkpoint inhibitors, may provide a new alternative option for TC with poor prognosis, as it has shown survival benefit in other oncology studies (21, 22). Currently, 12 immunotherapy trials have been registered in the ClinicalTrials.gov database and are recruiting patients.

DTC accounted for more than 90% of the new cases, and it is known to have a generally favorable prognosis, with a 5-year survival of >95% (23). It may be difficult and time-consuming to observe events such as recurrence or death; only a few pharmaceutical companies and investigators have conducted interventional studies for DTC patients and observed excellent long-term survival. The interventional studies included in the current study focused on patients with poor-prognosis TC subtypes of MTC, ATC, advanced or radioiodine-refractory DTC, as the treatment of these poor-prognosis TC subtypes remains a serious challenge. Among the studies registered in the ClinicalTrials.gov database, 35.3% of the interventional trials focused on poorer prognostic TC. Nonetheless, a worrisome and unfortunate limitation for conducting large phase III trials for MTC and ATC is the rarity of the diseases (23). Therefore, well-designed phase II randomized controlled trials for MTC and ATC may also be valuable.

Although registration in the ClinicalTrials.gov database has been associated with improved likelihood of publication, only 22.8–68% of completed studies registered in the ClinicalTrials.gov database were published (24–26). In the current study, 56.4% of the primary completed interventional studies before January 2017 were published. Typically, trials yielding negative results that do not favor or even contradict the initial hypotheses may be delayed or suppressed (27). In the current study, primary completed interventional studies after 2010 and those conducted in Asian countries were associated with improved publication likelihood. The public contribution of clinical studies may be lost when the results remain unpublished. Therefore, it is important to compel authors to publish their studies, even if they obtain negative outcomes, facilitating the publication of negative findings.

Among the published interventional studies about TC, the median time from primary completion to publication was 46.5 months. Only 52.3% of studies were published within 2 years since primary completion. Studies primary completed after 2010 and conducting in Asian had lesser time to publication. The time from primary completion to publication is quite long to some extent, and study results cannot be reported and introduced into clinical practice in time. Therefore, timely publication of trial results is a precondition for ensuring that physicians and other stakeholders make appropriate clinical decisions. Timely publication of trial results also reflects the best scientific evidence and yields maximum benefits for public health and scientific progress (24, 25). Encouragingly, the current study showed a trend of improvement in publication within 24 months compared with the studies completed before 2010. This possibly reflects the fact that investigators and sponsors attach increasing importance to timely sharing of trial results. Development in information technology may also have increased the efficiency and reduced the time needed from data collection to publication (27). Nevertheless, unremitting efforts need to continue to improve timely publication.

In the current study, an encouraging trend was that the number of both interventional and observational studies has been increasing in recent years. Similar to the studies for other oncological diseases (9, 28), clinical studies for TC are mostly small, single-institutional, and early-phase. The results of the current study revealed that early-phase studies were the most common type for TC, with phase II studies accounting for more than 42.3% of all interventional trials. Phase III randomized controlled trials are generally costly, time-consuming, and multi-center; therefore, starting and completing early-phase trials are logically easier than later-phase studies. Nonetheless, more well-designed phase III randomized controlled trials for TC are needed, as the results will play an irreplaceable role in changing clinical practice and decision-making in medicine. Another issue was that 60.1% of the interventional studies were registered after study initiation. As registration is expected to improve transparency in performing and reporting of studies, the rule that clinical trials should be registered before study initiation needs to be standardized.

This study has several limitations. The ClinicalTrials.gov does not include all clinical trials. Although many studies from other countries use the ClinicalTrials.gov database to satisfy the ICMJE registration requirements, seven other registries around the world may also be used (29). However, the ClinicalTrials.gov database still accounts for most clinical studies in the WHO portal. We relied on the ClinicalTrials.gov data provided by trial investigators or sponsors. In addition, the data sets for all trials in the database are not always complete and up to date. Moreover, we cannot exclude the possibility of errors owing to certain studies not being captured for the analysis and/or being potentially misclassified during the selection process. However, we took great care to minimize these limitations: two authors (YL and BL) crosschecked all identified studies and the trial selection steps. Furthermore, the NLM cannot verify the validity of all trial information, and some records might contain errors.

Overall, our study is the first to provide the current landscape of clinical studies about TC and indicated that high-level, randomized, phase 3 trials are still insufficient. Due to the indolent behavior of PTC and low incidence of poorer prognostic TC, it has been difficult to recruit and enroll patients in clinical trials in the past. More efforts are needed to promote the recruitment in the future. In addition, highly accurate methods are needed for the diagnosis of malignant nodules. Moreover, continued improvements in clinical trials are needed to improve the prognosis of aggressive cancers. Hence, more efforts are needed to promote the timely publication of clinical studies.

## Data Availability Statement

The raw data supporting the conclusions of this article will be made available by the authors, without undue reservation.

## Author Contributions

YL, BL, and QZ contributed equally to this article. HX and WL supervised the study. HX, YL, and BL designed the study. YL and QZ collected the data. BL and JX did the data analysis. YL, QZ, JL, FL, and BL wrote the draft report. SP performed critical revision on the manuscript. All authors contributed to the article and approved the submitted version.

## Funding

National Natural Science Foundation of China (81772850), Guangzhou Science and Technology Project (201803010057).

## Conflict of Interest

The authors declare that the research was conducted in the absence of any commercial or financial relationships that could be construed as a potential conflict of interest.
