# Supplementary material for: The Current Landscape of Clinical Studies Focusing on Thyroid Cancer: A Comprehensive Analysis of Study Characteristics and Their Publication Status
Source: Front Endocrinol (Lausanne). 2020 Nov 20;11:575799. doi: 10.3389/fendo.2020.575799 (PMC7714929; doi:10.3389/fendo.2020.575799)
Supplement: Supplementary file 1 [file Table_1.pdf]

Supplementary Table S1. Characteristic comparisons between IF groups among interventional published studies *

	Total (n=88)	IF < 10 (n=65)	IF ≥ 10 (n=23)	*P*-values
Duration of primary completion (mo.)	34.2 (20.5, 55.0)	33.9 (19.9, 56.0)	34.5 (28.0, 43.0)	0.618
Time to publication (mo.)	24.0 (11.2, 38.2)	24.7 (11.4, 42.0)	20.0 (11.1, 29.6)	0.176
Published within two years after primary completion				0.467
Within 2 years	42 (47.7)	33 (50.8)	9 (39.1)	
After 2 years	46 (52.3)	32 (49.2)	14 (60.9)	
Year of primary completion				0.610
00-10	27 (30.7)	19 (29.2)	8 (34.8)	
11-16	61 (69.3)	46 (70.8)	15 (65.2)	
Outcome				0.772
Nonpositive	18 (20.5)	14 (21.5)	4 (17.4)	
Positive	70 (79.5)	51 (78.5)	19 (82.6)	
Registered after study start				0.439
No	28 (31.8)	19 (29.2)	9 (39.1)	
Yes	60 (68.2)	46 (70.8)	14 (60.9)	
Sample size				0.776
<=50	44 (50.0)	31 (47.7)	13 (56.5)	
51-100	20 (22.7)	15 (23.1)	5 (21.7)	
>100	24 (27.3)	19 (29.2)	5 (21.7)	
Age				0.675
With child	8 (9.1)	7 (10.8)	1 (4.3)	
Only adults	80 (90.9)	58 (89.2)	22 (95.7)	
Only TC				0.228
No	18 (20.5)	11 (16.9)	7 (30.4)	
Yes	70 (79.5)	54 (83.1)	16 (69.6)	
Purpose				0.018
Others	13 (14.8)	13 (20.0)	0 (0.0)	
Treatment	75 (85.2)	52 (80.0)	23 (100.0)	
Phases				0.436
Phase 1	12 (13.8)	7 (10.9)	5 (21.7)	
Phase 2	38 (43.7)	27 (42.2)	11 (47.8)	
Phase 3/4	18 (20.7)	14 (21.9)	4 (17.4)	
NA	19 (21.8)	16 (25.0)	3 (13.0)	
Study design				0.330
Single group	48 (54.5)	33 (50.8)	15 (65.2)	
Two or more groups	40 (45.5)	32 (49.2)	8 (34.8)	
Randomization				0.325
No	52 (59.1)	36 (55.4)	16 (69.6)	
Yes	36 (40.9)	29 (44.6)	7 (30.4)	
Blind				1.000
Open label	65 (73.9)	48 (73.8)	17 (73.9)	

Blind	23 (26.1)	17 (26.2)	6 (26.1)	
Country				0.014
US/Canada	35 (39.8)	22 (33.8)	13 (56.5)	
European	19 (21.6)	17 (26.2)	2 (8.7)	
Asian	21 (23.9)	19 (29.2)	2 (8.7)	
Others	13 (14.8)	7 (10.8)	6 (26.1)	
Center				0.014
Single-center	54 (61.4)	45 (69.2)	9 (39.1)	
Multi-center	34 (38.6)	20 (30.8)	14 (60.9)	
Funder				0.012
NIH	17 (19.3)	12 (18.5)	5 (21.7)	
Industry	30 (34.1)	17 (26.2)	13 (56.5)	
Others	41 (46.6)	36 (55.4)	5 (21.7)	

Note: IF, impact factor; TC, thyroid cancer; NA, not available; NIH, the National Institutes of Health.

Supplementary Table S2. Multivariate logistic regression for high IF

	OR	95% CI	*P*-values
Country			
US/Canada	1.00		
European	0.09	(0.01, 0.85)	0.020
Asian	0.11	(0.01, 0.85)	0.034
Others	0.23	(0.03, 1.57)	0.123
Center			
Single-center	1.00		
Multi-center	6.55	(1.33, 32.30)	0.021
Funder			
Others	1.00		
NIH	0.58	(0.08, 4.24)	0.593
Industry	2.33	(0.58, 9.29)	0.232

Note: IF, impact factor; NIH, the National Institutes of Health.
